# Influence of left bundle branch block on left atrial strain parameters in patients with ischaemic and non-ischaemic dilated cardiomyopathy

**DOI:** 10.1007/s10554-026-03773-y

**Published:** 2026-07-21

**Authors:** Antonia Petersen, P. Kamieniarz, D. Meteva, T. Walter-Rittel, T. Elgeti, L.-A. Schaafs

**Affiliations:** 1 Department of Radiology, Charité - Universitätsmedizin Berlin, corporate member of Freie Universität Berlin and Humboldt-Universität zu Berlin, Hindenburgdamm 30, 12203 Berlin, Germany; 2 Department of Cardiology, Angiology and Intensive Care Medicine, Charité - Universitätsmedizin Berlin, corporate member of Freie Universität Berlin and Humboldt-Universität zu Berlin, Hindenburgdamm 30, 12203 Berlin, Germany; 3 Department of Radiology, Charité – Universitätsmedizin Berlin, corporate member of Freie Universität Berlin and Humboldt-Universität zu Berlin, Luisenstraße 10, 10117 Berlin, Germany

**Keywords:** Cardiac magnetic resonance imaging, Dilated cardiomyopathy, Left bundle branch block, Left atrial strain

## Abstract

Left atrial (LA) strain parameters predict mortality in patients with ischaemic/non-ischaemic dilated cardiomyopathy (DCM). Left bundle branch block (LBBB) is a common conduction abnormality in those patients. To date, the impact of LBBB on LA strain parameters assessed by cardiovascular magnetic resonance imaging (cMRI) has not been studied systematically. In this retrospective single-centre study, we included 20 (10 female) controls without DCM or LBBB, 21 (11 female) patients with LBBB without DCM, 20 (7 female) patients with DCM without LBBB, and 20 (9 female) patients with DCM and LBBB. LA volumes and strain were assessed semi-automatically using the cvi42^®^ and Segment^®^ software. LA reservoir/conduit strain and corresponding strain rates were significantly impaired in patients with DCM (*p* = 0.003/0.022 respectively < 0.001/0.004) in comparison to controls. The presence of LBBB did not lead to significant changes of LA reservoir/conduit strain in controls (*p* = 0.919/0.391) nor in patients with DCM (*p* = 0.862/0.999), same was true for LA reservoir/conduit strain rate in patients with DCM (*p* = 0.999/0.812). LA conduit strain rate of patients with LBBB was significantly impaired in comparison to controls (*p* = 0.005). Left ventricular dilatation was associated with significantly impaired LA reservoir/conduit strain/strain rates. The presence of LBBB was not associated with additional impairment in LA strain/strain rate parameters in patients with ischaemic/non-ischaemic DCM, supporting the robustness of LA functional parameters in the presence of this common conduction abnormality. However, in patients with LBBB without dilatation LA conduit strain rate was significantly impaired in comparison to controls and further studies are needed to evaluate the impact of this finding.

**Graphical abstract:**

**Figure:**
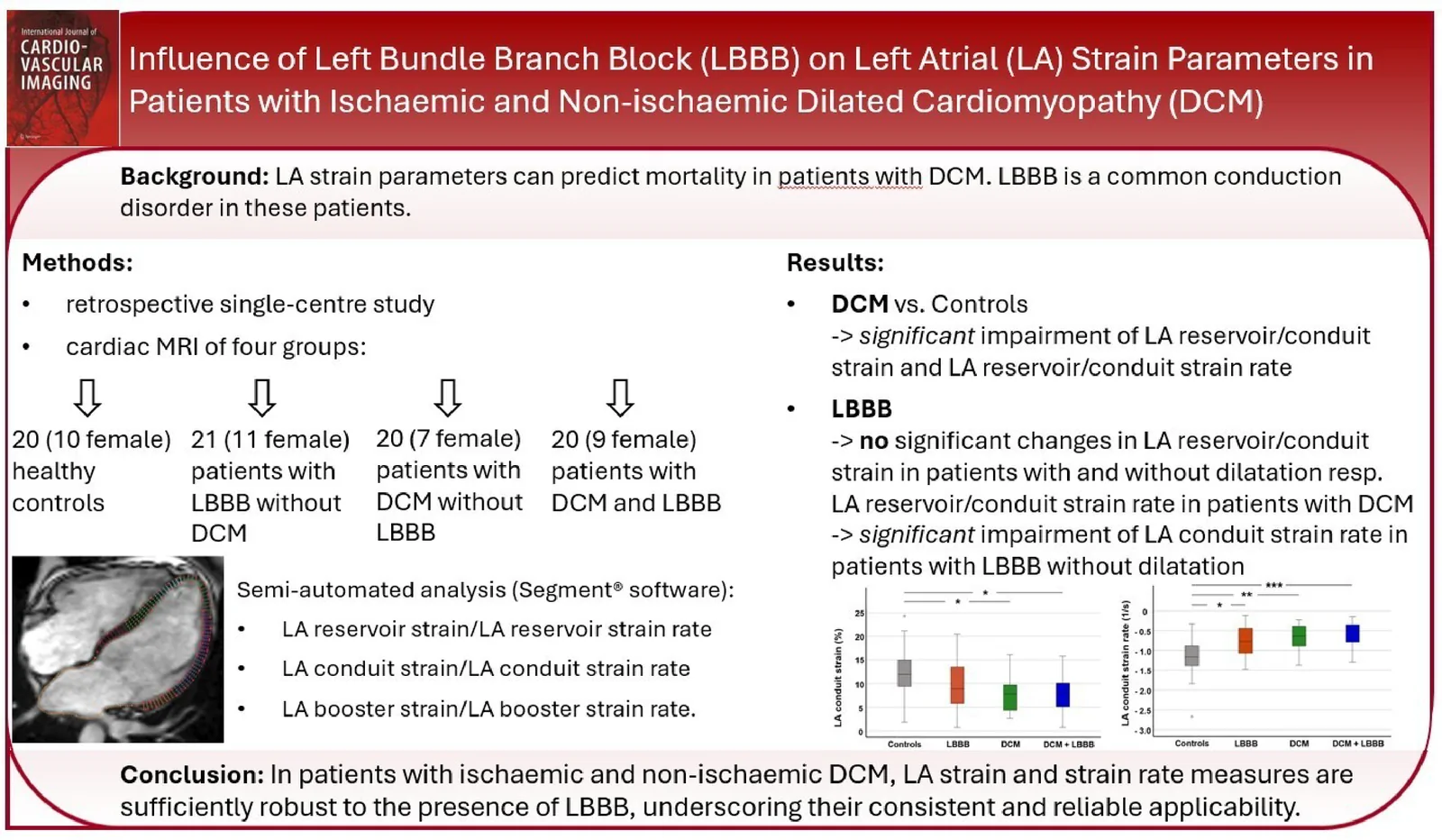

## Introduction

Dilated cardiomyopathy (DCM) is one of the common phenotypes of cardiomyopathy and is characterized by the dilatation of the left ventricle and impaired left ventricular (LV) systolic function without presence of coronary artery disease or other secondary causes sufficient to explain ventricular remodeling. Late-stage presentation of other non-ischemic and ischemic heart diseases share the dilated phenotype [1, 2]. Patients with DCM carry a considerable risk for adverse cardiac events, which is why risk stratification plays an important role in therapy planning [3].

Left bundle branch block (LBBB) is a common conduction disorder with a reported prevalence of up to 30% in patients with DCM [4–6]. LBBB is characterized by a delay in the conduction of the LV excitation and thus dyssynchronous LV contraction [7]. Due to atrioventricular coupling, LV dyssynchrony may also affect atrial mechanics, with echocardiographic studies describing time-delayed motion of the left atrial (LA) lateral wall relative to the LA septal wall in patients with LBBB and heart failure, and partial reversal after cardiac resynchronisation therapy (CRT) in responders [8–11].

In the recent years, LA size and function have become the subject of scientific debate with regard to its prognostic value in patients with DCM, as it provides information about both systolic and diastolic function of the left ventricle [12, 13]. Strain analysis using feature tracking (FT) in cardiac magnetic resonance imaging (cMRI) has proven to be an accurate and reproducible method for analysing LA phase function. The LA reservoir strain represents the maximum passive filling of the LA until the end of LV systole, the LA conduit strain the passive emptying of the LA during LV early- to mid-diastole, and the LA booster strain the active contraction of the LA at LV end-diastole [14–16]. In particular reservoir and conduit strain were found to be independent prognostic parameters of adverse clinical outcomes in patients with non-ischaemic and ischaemic DCM [12, 17, 18]. The same applies for the corresponding strain rates [19].

To our best knowledge the influence of LBBB on LA strain parameters derived from cMRI has not been systematically examined. Therefore, this study aimed to compare LA strain and strain rate parameters in patients with ischaemic and non-ischaemic DCM with and without LBBB. Controls without any pathological findings on cMRI and patients with LBBB without LV dilatation served as comparison groups.

## Materials and methods

### Study population

This is a retrospective, single-centre study approved by the internal review board (IRB) of our institution (application number: EA2/237/21). Informed consent was waived due to the retrospective design of the study. All patients with LBBB with or without DCM examined between 2015 and 2023, complemented by consecutive patients with DCM without LBBB examined in 2024, who underwent cMRI at our institution and met the inclusion criteria were included in this study. Age- and sex-matched cMRI examinations without pathological findings on imaging or subsequent work-up served as controls; these scans were performed for non-specific cardiac symptoms, such as suspected, though not otherwise proven myocarditis or non-specific chest pain. Inclusion criteria were age > 18 years and complete cMRI datasets. The diagnosis of LBBB was made according to the 2021 European Society of Cardiology criteria, i.e., when the ECG showed a widened Q wave, R wave, S wave (QRS) complex of > 120 ms and any other ECG characteristics of LBBB [20]. DCM was defined as increased LV end-diastolic volume (LVEDV) and reduced LV ejection fraction (LVEF) in relation to nomograms [21]. Exclusion criteria were congenital heart disease, acute myocarditis, severe valvular disease, right bundle branch block and cardiac resynchronization therapy. All patients were in sinus rhythm. The clinical parameters were obtained from the patients` records. cMRI datasets from the years 2015–2023 overlap with the study population of two previous studies (doi: https://doi.org/10.3389/fcvm.2022.977414, https://doi.org/10.1038/s41598-024-55821-z) with focus on LV dyssynchrony and T1 mapping. The clinical parameters were obtained from the patients` records. This study adheres to the Sex and Gender Equity in Research (SAGER) guidelines.

### Image acquisition

cMRI was performed on a 1.5T MRI system (Magnetom Aera, Siemens Healthineers, Erlangen, Germany). CINE-images were acquired using retrospectively gated 2D steady-state free precession (SSFP) pulse sequences in standard long-axis (2-, 3-, 4-chamber) and contiguous short-axis stacks covering the LV (typical parameters: TR 34 ms, TE 1.29 ms, flip angle 5°, in plane resolution of 1.7 mm x 1.7 mm, slice thickness of 5 mm for long-axis acquisition, 8 mm with 2 mm interslice gap for short-axis acquisition, reconstructed temporal resolution 35–44 ms). LGE imaging was performed 10–12 min after administration of 0.15 mmol/kg Gadobutrol (Gadovist^®^, Bayer AG, Leverkusen, Germany) in long-axis (2-, 3-, 4-chamber) in short-axis slices using an inversion-recovery-prepared T1-weighted gradient echo sequence with a manually adjusted inversion time (TI) based on the TI scout as well as a parallel utilized phase-sensitive inversion-recovery (PSIR)-based reconstruction algorithm (typical parameters: TR 920 ms, TE 3.3 ms, TI 270 ms, flip angle 25°, FOV 360 mm x 292 mm, matrix 256 × 156, pixel spacing 1.45 mm x 1.45 mm, slice thickness 8, 2 mm interslice gap).

### Image analysis

**Fig. 1:**
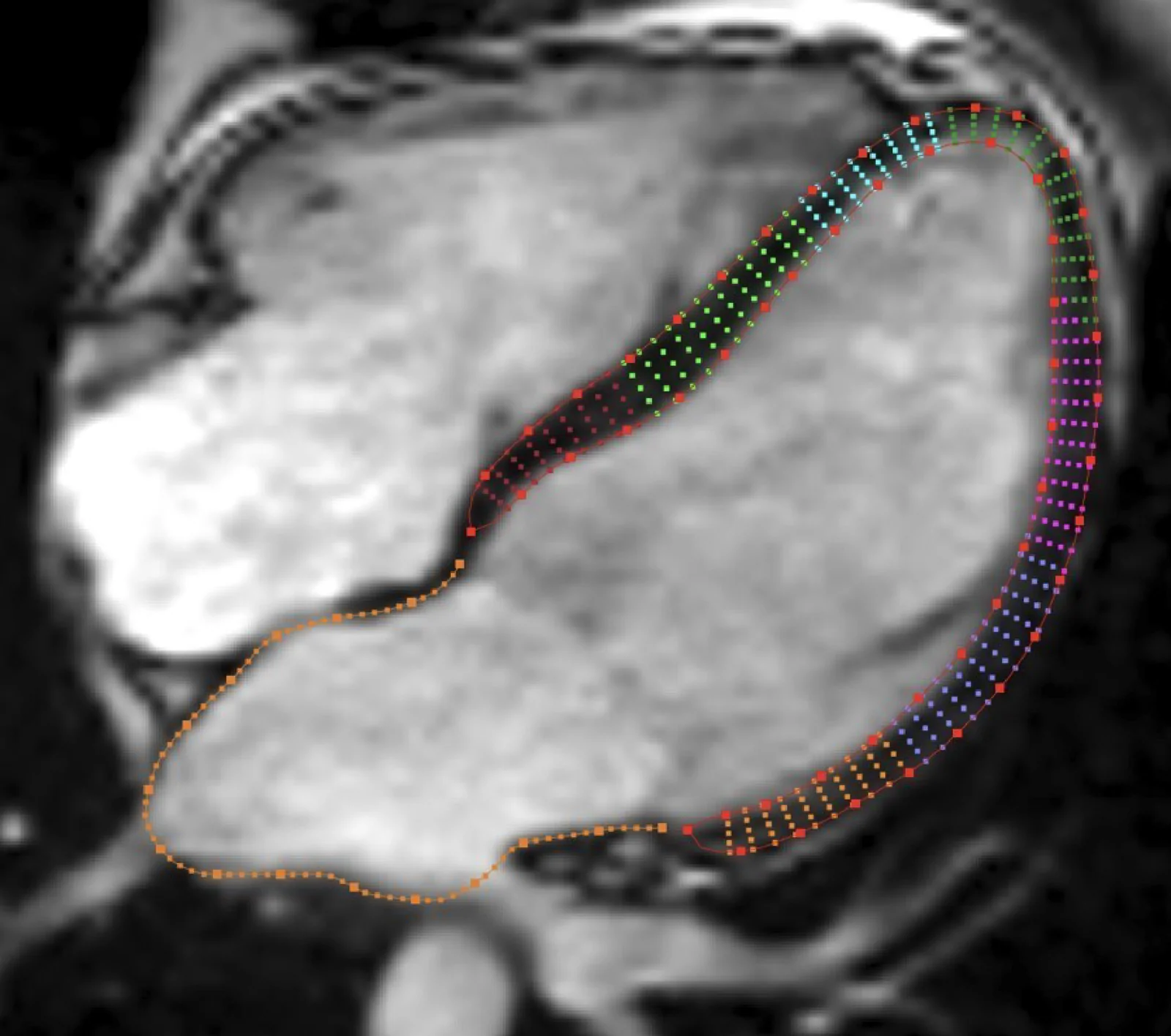
4-chamber long-axis CINE view. The orange dotted line delineates the contour of the left atrium in left ventricular end-diastole, the red dotted line delineates the contour of the left ventricle. Both contours were semi-automatically generated using the Segment^®^ software and manually corrected

cMRI datasets were evaluated by three board-certified radiologists with > 5 (A.P.), > 10 (L-A.S.) respectively > 15 years (T.E.) of experience. Volume and strain analysis was carried out by one radiologist (A.P.), LGE images were evaluated in consensus for the presence and distribution of LGE by two radiologists (L.-A. S., T.E.). The analysis of the LA and LV volumes was carried out using the cvi42^®^ software (Release 5.14, Circle Cardiovascular Imaging, Calgary, Canada). The analysis of LV volumes (LVEDV, LV end-systolic volume/LVESV, LV stroke volume/LVSV, LVEF) was performed in CINE short-axis views. Endo- and epicardial contours were drawn semi-automatically and corrected manually if necessary. The analysis of LA volumes was performed accordingly in the CINE 4-chamber view. LV and LA volume values were indexed to the body surface area (BSA). Strain analysis was done using the Segment^®^ software (v.4.1, Medviso, segment.heiberg.de). LV global longitudinal strain (LVGLS), LA reservoir strain, LA conduit strain and LA booster strain and corresponding LA strain rates, LA reservoir strain rate, LA conduit strain rate, LA booster strain rate, were obtained in CINE 4-chamber views. LV and LA contours were drawn semi-automatically and corrected manually if necessary. An example is shown in Fig. 1. cMRI from patients that could not be evaluated properly due to artifacts were excluded (*n* = 4). After three months LA strain analysis of 20 cMRI was repeated by one radiologist (A.P.) to ensure that a high level of reproducibility had been achieved [22]. The same subset of 20 cMRI examinations was independently re-evaluated by a second radiologist (P.K.) to assess interrater reliability.

### Statistical analysis

IBM SPSS Statistics^®^ 28.0.1.0 (IBM Corporation, Armonk, New York, United States) was used for statistical analysis. Given the small group sizes, a non-normal distribution was expected; therefore, age, QRS width, LA and LV volume, and strain values were evaluated using the non-parametric Kruskal-Wallis test with subsequent pairwise group comparisons performed via the Steel-Dwass test. A two-way ANOVA was added to evaluate the main and interaction effects of LA dilatation and LBBB on LA strain and strain rate parameters. A chi-square test was conducted to compare binarized variables. The correlations between LA strain and strain rate values and QRS width was calculated using Spearman`s rank correlation coefficient (ρ). Intra- and inter-rater agreement for the analysis of LA Strain values was determined using the intraclass correlation coefficient (two-way random, absolute agreement). To evaluate statistical test power, a retrospective post-hoc power analysis was performed for all LA strain respectively strain rate parameters using G*Power software (version 3.1.9.7). To account for non-parametric testing, calculations were based on a one-way ANOVA model and adjusted assuming a 95.5% asymptotic relative efficiency (ARE) for the Kruskal-Wallis test [23]. The calculation was based on an α-level of 0.05 and the observed effect sizes (Cohen’s f) derived from the Welch-ANOVA variance model. Descriptive parameters are given as median and inter-quartile-range (IQR) or mean ± standard deviation depending on whether normal distribution could be assumed. A p-value < 0.05 was considered statistically significant.

## Results

### Cohort

A summary of demographic data, clinical characteristics and presence of LGE can be found in Table 1. Table 2 summarizes the medications taken by the patients. A total of 61 patients (27 female) and 20 controls (10 female) were included, of these 21 patients (11 female) with LBBB, 20 patients with DCM without LBBB (7 female) and 20 patients with DCM with LBBB (9 female). Median age was 55 years (interquartile range/IQR 24 years). Median heart rate was 65 beats per minute (bpm) (IQR 22 bpm). There were no significant group differences in terms of gender distribution, age and heart rate (*p* = 0.693/0.241/0.674). Median QRS width in controls was 94 ms (IQR 16 ms) and 90 ms (IQR 10 ms) in patients with DCM without LBBB. In patients with LBBB median QRS width was 138ms (IQR 33 ms) and 154 ms (IQR 21 ms) in patients with DCM and LBBB. There was no significant difference in QRS width between patients with LBBB and patients with DCM and LBBB (*p* = 0.184).

**Table 1:** Clinical characteristics

	Controls	LBBB	DCM	DCM+ LBBB	*p*-value
*n* (female)	20 (10)	21 (11)	20 (7)	20 (9)	0.693
Age (years)	57 (29)	66 (21)	51 (25)	58 (13)	0.241
Heart rate (beats per minute)	59 (11)	63 (25)	66 (26)	66 (18)	0.674
QRS width (ms)	94 (16)	138 (33)	90 (10)	154 (21)	< 0.001
Coronary artery disease (n)	0	1	5	4	0.047
Hypertension (n)	4	7	7	10	0.262
Smoking (n)	2	4	3	2	0.818
Hyperlipidaemia (n)	7	2	5	7	0.599
Obesity (n)	1	2	1	2	0.880
Diabetes (n)	1	1	1	3	0.158
LGE total	0	7	10	12	< 0.001
LGE ischaemic	0	3	3	6	0.055
LGE non-ischaemic	0	4	7	6	0.015

Parameters are given as median and inter-quartile-range (IQR)

**Table 2:** Medication use

	Controls	LBBB	DCM	DCM+ LBBB	*p*-value
β-blocker (*n*)	5	11	14	16	0.003
ACE inhibitor/ARB (n)	4	11	12	18	0.001
Diuretic (n)	1	6	4	9	0.025
Spironolactone (n)	0	2	8	10	0.001
Calcium channel blocker (n)	4	4	2	3	0.909
Digoxin (n)	0	0	0	0	
SGLT-2 inhibitor (n)	1	4	11	4	0.003
DPP4 inhibitor (n)	0	0	0	1	0.741
Metformin (n)	1	0	0	2	0.323
Sulfonylurea (n)	1	0	0	0	0.741
Insulin (n)	2	0	1	2	0.472
Aspirin (n)	2	5	8	8	0.108
NOAC therapy (n)	0	2	4	7	0.011
Warfarin (n)	0	0	0	0	
Statin (n)	6	8	5	9	0.569

n: number of patients per group. ACE = angiotensin-converting enzyme, ARB = angiotensin receptor blocker, NOAC = non-vitamin-K oral anticoagulant, SLGT-2 = sodium-glucose cotransporter 2, DPP4 = dipeptidyl peptidase IV

### LA and LV volume and LV GLS analysis

Table 3 provides a comprehensive overview of indexed LA and LV volume parameters. LVEDV/BSA, LVESV/BSA, LVEF and LVGLS were significantly lower in controls than in patients with DCM (*p* = 0.003/< 0.001/< 0.001/0.003) and patients with DCM and LBBB (p = < 0.001/< 0.001/< 0.001/0.001). This also applied to patients with LBBB compared to patients with DCM (*p* = 0.002/0.002/0.023) and patients with DCM and LBBB (p = < 0.001/< 0.001/0.001) in terms of LVEDV/BSA, LVESV/BSA and LVEF. Since LVGLS is expressed in negative percentages, superior function is represented by more negative (numerically lower) values.

**Table 3:** LV and LA function parameters

	Controls	LBBB	DCM	DCM + LBBB	*p*-value
LVEDV/BSA (ml/m^2^)	87.87 (18.50)Ɨ^	89.16 (35.97)Ɨ^	109.40 (27.04)	137.25 (38.42)	< 0.001
LVESV/BSA (ml/m2)	31.41 (13.34)*Ɨ^	45.38 (12.71)Ɨ^	66.18 (37.12)	87.87 (48.01)	< 0.001
LVSV/BSA (ml/m2)	56.86 (10.16)*	43.57 (20.30)	46.59 (23.90)	52.14 (16.81)	0.018
LVEF (%)	61.59 (5.10)*Ɨ^	50.15 (14.85)Ɨ^	38.17 (23.26)	37.43 (17.81)	< 0.001
LVGLS (%)	-19.19 (4.12)*Ɨ^	-15.69 (4.91)	-9.15 (10.40)	-12.15 (5.98)	< 0.001
LVMASS/BSA (g/m2)	58.80 (11.81)Ɨ^	66.22 (18.92)^	75.51 (18.09)^	90.53 (32.09)	< 0.001
Minimal LA VOL/BSA (ml/m^2^)	17.25 (9.74)	11.92 (8.31)Ɨ^	19.95 (17.92)	16.79 (24.35)	0.002
Maximal LA VOL/BSA (ml/m^2^)	39.55 (15.08)*	31.88 (18.20)Ɨ^	41.90 (22.85)	37.93 (29.88)	0.008
LA EF (%)	60.63 (18.96)Ɨ	56.94 (13.99)Ɨ	48.81 (22.99)	55.70 (21.29)	0.001
LA reservoir strain (%)	18.23 (10.82) 1Ɨ	14.02 (14.07)	10.71 (7.72)	11.43 (8.92)	0.003
LA conduit strain (%)	11.91 (5.82)Ɨ^	8.89 (8.94)	7.79 (5.85)	8.04 (5.23)	0.017
LA booster strain (%)	6.49 (5.91)	6.87 (9.55)Ɨ	3.10 (5.19)	3.84 (5.76)	0.035
LA reservoir strain rate (1/s)	0.80 (0.39)Ɨ^	0.74 (0.65)	0.50 (0.30)	0.52 (0.38)	0.001
LA conduit strain rate (1/s)	-1.17 (0.56)*Ɨ^	-0.77 (0.65)	-0.65 (0.54)	-0.50 (0.44)	< 0.001
LA booster strain rate (1/s)	-0.77 (0.52)Ɨ^	-0.79 (0.68)	-0.50 (0.45)	-0.50 (0.48)	0.005

Parameters are given as median and inter-quartile-range (IQR)

* vs. LBBB, Ɨ vs. DCM, ^ vs. DCM + LBBB with *p* < 0.05

Minimal LA Vol/BSA showed no significant difference between controls and patients with LBBB (*p* = 0.414) and patients with DCM and patients with DCM and LBBB (*p* = 0.977). Maximal LA Vol/BSA was significantly smaller in controls than in patients with LBBB (*p* = 0.035), but there was no significant difference between patients with DCM and patients with DCM and LBBB (*p* = 0.982). There was also a significant difference between patients with LBBB and patients with DCM (*p* = 0.007/0.042), respectively patients with DCM and LBBB (*p* = 0.034/0.027) regarding both values. LAEF was significantly lower in patients with DCM than in controls (*p* = 0.002) and patients with LBBB (*p* = 0.031).

### LA strain analysis

**Fig. 2:**
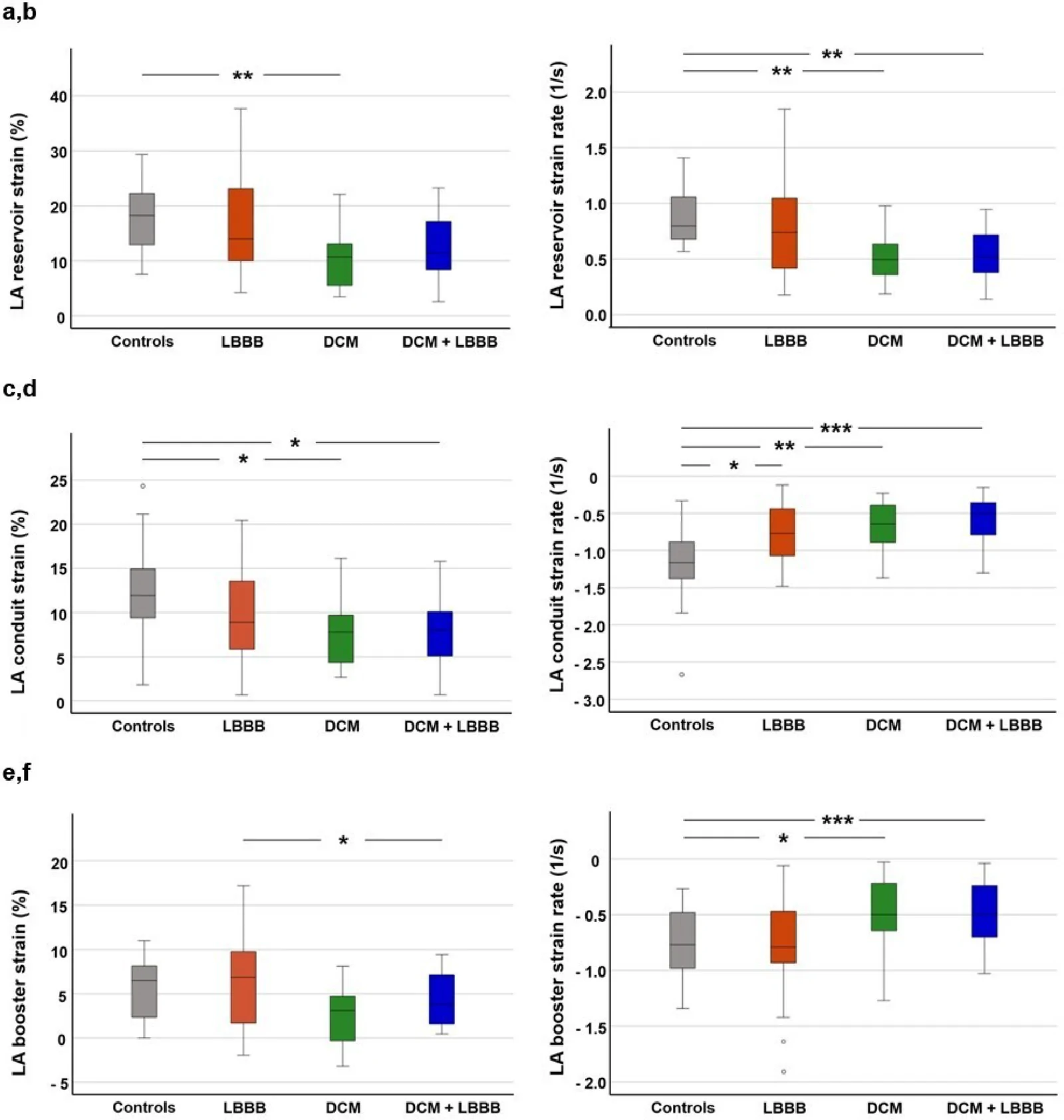
Comparison of left atrial (LA) strain and LA strain rate values between controls, patients with left bundle branch block (LBBB), patients with dilated cardiomyopathy (DCM) and patients with DCM and LBBB. **a**: LA reservoir strain, **b**: LA reservoir strain rate, **c**: LA conduit strain, **d**: LA conduit strain rate, **e**: LA booster strain, **f**: LA booster strain rate. Asterisks indicate a statistically significant difference with *p < 0.05, **p < 0.01, ***p < 0.001

**Fig. 3:**
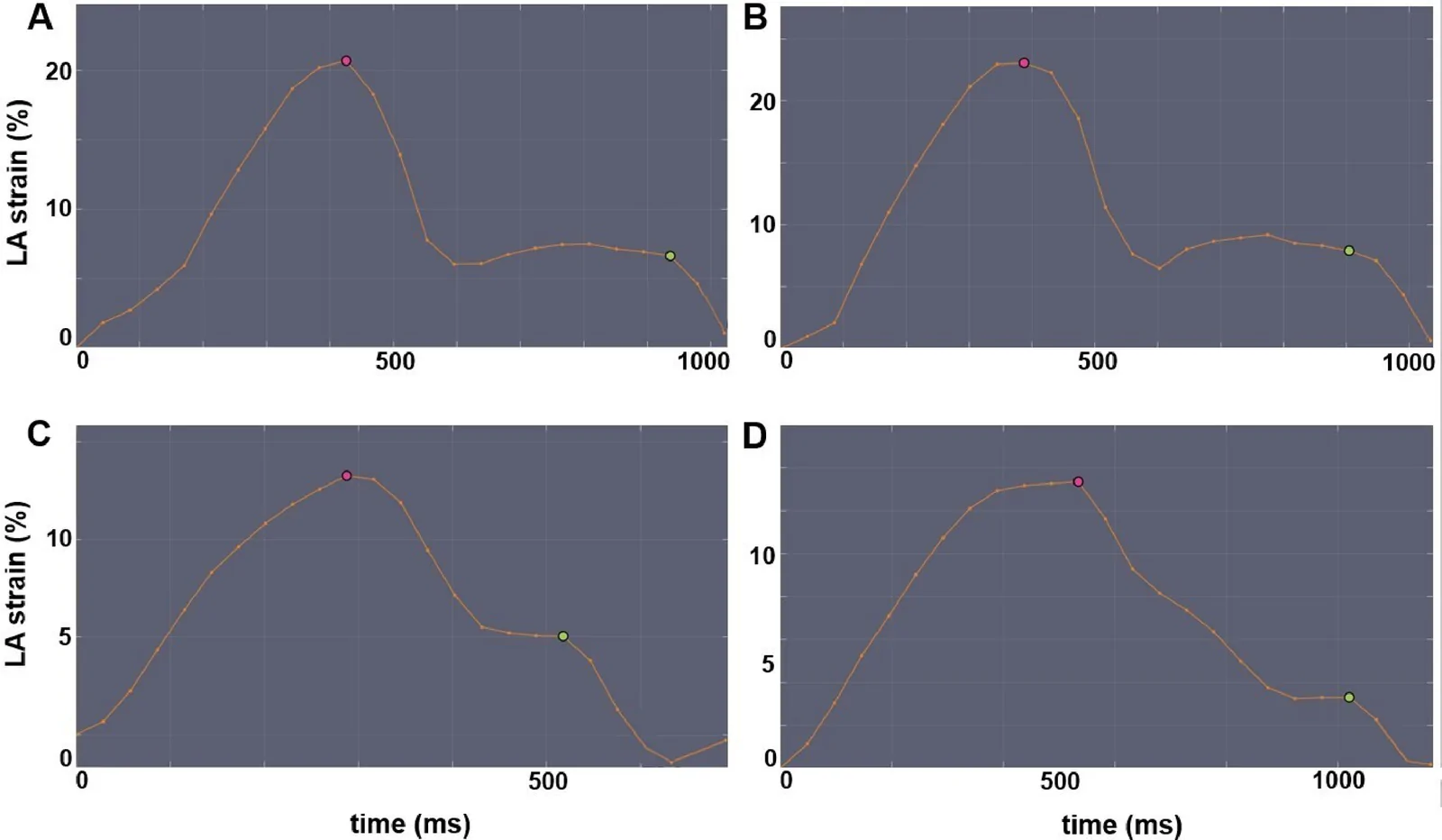
Representative left atrial (LA) strain curves from a **A**: control, **B**: patient with left bundle branch block (LBBB), **C**: patient with dilated cardiomyopathy (DCM), **D**: patient with DCM and LBBB. The x-axis shows time in milliseconds (ms), the y-axis shows LA strain values in percent (%). The pink dot indicates LA reservoir strain, the green dot indicates LA booster strain, LA conduit strain is calculated as the difference between LA reservoir strain and LA booster strain. Significant changes in LA reservoir and LA conduit strain are observed in patients with DCM, while the presence of LBBB has no significant effect

LA strain parameters are summarized in Table 3 and depicted as boxplots in Fig. 2. There were no significant differences in LA reservoir, LA conduit and LA booster strain values between controls and patients with LBBB (*p* = 0.919/0.391/0.871) nor between patients with DCM and patients with DCM and LBBB (*p* = 0.862/0.999/0.621). LA reservoir strain was significantly lower in patients with DCM (*p* = 0.003) than in controls. LA conduit strain was significantly lower in patients with DCM (*p* = 0.022) and patients with DCM and LBBB (*p* = 0.025) than in controls. LA booster strain was significantly lower in patients with DCM than in patients with LBBB (*p* = 0.041). Representative strain curves of controls, patients with LBBB, patients with DCM and patients with DCM and LBBB are shown in Fig. 3.

LA reservoir, conduit and booster strain were significantly associated with LA dilatation (*p* < 0.001/0.003/0.004), but not with LBBB (*p* = 0.923/0.315/0.162), with no significant interaction (*p* = 0.235/0.230/0.588).

Intra-observer agreement for LA strain parameters were excellent with intra-class correlation coefficients ranging from 0.986 (95% confidence interval of 0.964–0.994) and 0.988 (95% confidence interval of 0.969–0.995). Inter-observer agreement for LA strain parameters was also excellent with intra-class correlation coefficients ranging from 0.958 (95% confidence interval of 0.829–0.986) and 0.989 (95% confidence interval of 0.971–0.996).

### LA strain rate analysis

LA strain rate parameters are summarized in Table 3 and depicted as boxplots in Fig. 2. LA reservoir, LA conduit strain and LA booster strain rate were significantly higher respectively lower in controls than in patients with DCM (*p* = 0.001/0.001/0.019) and patients with DCM and LBBB (*p* = 0.001/< 0.001/0.014). LA conduit strain of controls was additionally significantly lower in controls than in patients with LBBB (*p* = 0.005). Otherwise, there were no significant differences between controls and patients with LBBB nor between patients with DCM and patients with DCM and LBBB in terms of LA strain rates. Since LA conduit and LA booster strain rate are expressed in negative values, superior function is represented by more negative (numerically lower) values. LA reservoir and booster strain rate were significantly associated with LA dilatation (*p* < 0.001/0.001), but not with LBBB (*p* = 0.586/0.953), with no significant interaction (*p* = 0.254/0.871). LA conduit strain rate was significantly associated with both, LBBB (*p* = 0.009) and LA dilatation (*p* < 0.001), with no significant interaction (*p* = 0.062).

### Correlation analysis

There was no significant correlation between QRS width and LA reservoir strain (ρ = 0.072, *p* = 0.600), LA conduit strain (ρ = 0.023, *p* = 0.867) and LA booster strain (ρ = 0.115, *p* = 0.398). There was also no significant correlation between QRS width and LA reservoir strain rate (ρ = -0.132, *p* = 0.332), LA conduit strain rate (ρ = 0.198, *p* = 0.143) and LA booster strain rate (ρ = 0.081, *p* = 0.553).

### Post-Hoc power analysis

Retrospective post-hoc power analysis showed a strong statistical power for LA reservoir strain (1 – β = 0.823), LA reservoir strain rate (1 – β = 0.933), LA conduit strain (1 – β = 0.937), and LA conduit strain rate (1 – β = 0.918). A moderate statistical power was observed for LA booster strain (1 – β = 0.749), whereas the power for LA booster strain rate remained low (1 – β = 0.326).

## Discussion

In this study LA reservoir strain was significantly lower in patients with DCM without LBBB than in the control group, the same was true for LA conduit strain in patients with DCM with and without LBBB. The presence of LBBB wasn`t accompanied by any significant changes in LA reservoir, LA conduit and LA booster strain values in controls nor in patients with DCM. LA conduit strain rate of the control group was significantly lower than in patients with LBBB, otherwise there were no significant differences between controls and patients with LBBB nor between patients with DCM without and with LBBB in terms of LA reservoir, conduit and booster strain rate. LA reservoir, conduit and booster strain were significantly associated with LA dilatation, but not with LBBB, and there was no significant interaction between LA dilatation and LBBB.

The measured LA strain values of controls and patients with DCM in our study are broadly consistent with previously reported ranges in the literature [17, 18, 24]. In our study there were no significant differences between all groups in terms of gender distribution, age and heart rate. The measurement of LA strain parameters could be reproduced with excellent reliability. LA strain values are representative of LV systolic and diastolic function and are considered more sensitive with regard to pathological conditions than LA volume or area measurements, which is in line with the assessed values of our study population [25, 26]. In our cohort, LA reservoir and conduit strain values were significantly lower in patients with DCM than in controls, while LA volumes/BSA did not show any significant differences.

Dokuni et al. suggest that in patients with LBBB the early contraction of the interventricular septum and the subsequent contraction of the LV lateral wall are transmitted one-to-one to the septal and lateral walls of the LA, respectively, thereby causing dyssynchronous stretching of the left atrium while LV contraction [8]. However, in our study, the presence of LBBB did not lead to a significant change in LA strain values during systole nor during the rest of the cardiac cycle in controls nor in patients with DCM. This is in line with the echocardiographic findings of Parks et al., who found an elevated LA dyssynchrony index in patients with non-ischaemic dilated cardiomyopathy in the presence of LBBB but no significant differences in LA reservoir strain values [27]. LA conduit and booster strain and strain rate values were not evaluated in that study.

CRT therapy in patients with LBBB was able to significantly increase LA reservoir strain values, yet this was only true for responders to CRT therapy [28]. In our study there was no significant correlation between QRS width and LA strain values, however patients with an indication for CRT were not part of this study.

LA conduit strain rate was significantly lower in patients with LBBB than in the control group in our patient cohort and was significantly associated with LBBB in the two-way ANOVA. Otherwise, LA strain rate values didn`t show any significant differences between controls and patients with LBBB nor in patients with DCM without and with LBBB. Patients with LBBB show an elevated end-diastolic LV pressure, which might lead to the observed difference in LA conduit strain rates between controls and patients with LBBB [29]. This effect might have been outweighed by other mechanical respectively hemodynamic factors in patients with DCM without and with LBBB. Still, it might play a role during exercise, as end-diastolic LV pressure in patients with LBBB is further increased during tachycardia [30].

Several studies have investigated cMRI-derived LA strain parameters in terms of their predictive value in patients with DCM without evaluating the influence of LBBB on LA strain and strain rate parameters in general [12, 17]. Li et al. identified LA reservoir and conduit strain as independent predictors of an adverse outcome in patients with idiopathic DCM, however LBBB wasn`t identified as a discriminating factor, which is in line with our findings [31].

Several limitations have to be considered: This is a single-centre study with a small sample size of 20 respectively 21 patients per group and the lack of an a priori sample size calculation. While post-hoc power analysis confirmed a strong statistical power for LA reservoir/conduit strain respectively strain rate parameters and a moderate statistical power for LA booster strain, LA booster strain rate was underpowered, indicating a substantial 0.659 risk of a Type II error. LA strain analysis was solely performed in the 4-chamber view; which might underestimate absolute LA strain values [32, 33]. No regional LA strain analysis was performed to address the dyssynchronous movement pattern in patient with LBBB. The control group consists of individuals who underwent cMRI due to non-specific thoracic symptoms, therefore imaging-occult alterations that influence LV/LA functional and strain parameters cannot be ruled out entirely. Due to the small population and the retrospective study design it was also not possible to form further subgroups, e.g. to evaluate patients with ischaemic and non-ischaemic DCM separately. An analysis stratified by sex was not feasible, though the study groups did not differ significantly regarding sex distribution. No uniform follow-up data were available to include a prognostic analysis.

## Conclusion

In this study, we investigated whether LBBB has an influence on LA strain values in patients with and without LV dilatation. We found that in patients with ischaemic and non-ischaemic DCM, LV dilatation and dysfunction were associated with a significant impairment of LA reservoir and conduit strain as well as corresponding strain rates regardless of the presence of LBBB. Similarly, LBBB did not lead to a significant decrease in LA reservoir and conduit strain values in patients without LV dilatation. Taken together, in patients with ischaemic and non-ischaemic DCM, LA strain and strain rate measures show sufficient robustness to the presence of LBBB as a common conduction abnormality in these patients underscoring their consistent and reliable applicability as functional markers. However, we found significantly lower LA conduit strain rates in patients with LBBB compared to controls and further studies are needed to investigate the implications of this result.

## Data Availability

The datasets used and/or analysed during this current studyare available from the corresponding author on reasonablerequest.
